# Inclisiran, Reasons for a Novel Agent in a Crowded Therapeutic Field

**DOI:** 10.1007/s11883-024-01271-x

**Published:** 2025-01-09

**Authors:** Francesco Di Giacomo-Barbagallo, Natalia Andreychuk, Roberto Scicali, Ana Gonzalez-Lleó, Salvatore Piro, Lluis Masana, Daiana Ibarretxe

**Affiliations:** 1 Unitat de Medicina Vascular I Metabolismo, Hospital Universitario Sant Joan, Universitat Rovira I Virgili, IISPV, CIBERDEM, Reus, Spain; 2 Department of Clinical and Experimental Medicine, Internal Medicine, Garibaldi-Nesima Hospital, University of Catania, 95122 Catania, Italy; 3 Faculty of Medicine, Universitat Rovira I Virgili, C/ Sant Llorenç, 21 43201, Reus, Spain

**Keywords:** Inclisiran, Lipid lowering therapy, LDLcholesterol, Cardiovascular prevention, PCSK9 targeted therapies

## Abstract

**Purpose of the Review:**

A significant number of patients fail to achieve target LDL cholesterol (LDL-C) levels. This review aims to explore why inclisiran, a novel class of LLT, should be considered a valuable addition to the current treatment options.

**Recent Findings:**

Inclisiran is a small interfering RNA (siRNA) that targets PCSK9 synthesis specifically in the hepatocytes. The drug remains in circulation for less than 48 h, but its effect lasts for over six months. Two subcutaneous injections per year consistently lowers LDL-C by approximately 55% with a favorable safety profile. In combination with other LLTs, it can achieve LDL-C reductions of over 80%, supporting its role in high-intensity LLT strategies.

**Summary:**

Inclisiran represents a novel class of LLT. Administered biannually, reduces baseline LDL-C levels by half. Additionally, it has a strong safety profile. Due to its pharmacokinetic properties, is likely to improve adherence to LLT and persistently maintain low LDL-C levels.

## Introduction

Cardiovascular diseases (CVD) are the leading cause of mortality and morbidity worldwide. According to data from the European Society of Cardiology, approximately 40% of all deaths are due to CVD in both men and women, representing twice the number of deaths caused by all forms of cancer and neurodegenerative diseases combined. Moreover, CVD also constitutes the primary cause of disability, accounting for a significant loss in quality-adjusted life years [1].

Atherosclerotic CVD (ASCVD) is the predominant cause among all CVDs. This condition results from the accumulation of cholesterol in the intimal layer of arteries, triggering an inflammatory response that leads to the formation of atherosclerotic plaques [2].

The causal role of cholesterol in the development of ASCVD is beyond any doubt. An overwhelming amount of epidemiological, animal experimental, genetic, and clinical data establishes the etiological role of cholesterol transported by apo B-containing lipoproteins, primarily low-density lipoprotein (LDL). Additionally, plasma concentrations of LDL cholesterol (LDL-C) and cholesterol contained in apo B-carrying lipoproteins are directly associated with the risk of ASCVD [3].

The evidence that lowering LDL-C levels is associated with a reduction in CVD is highly robust. Results from randomised controlled trials (RCTs) exploring the effect of lipid-lowering therapies (LLT), involving several hundreds of thousands of patients, have established that regardless of the drug used, a reduction of 1 mmol/L in LDL-C is associated with approximately a 22% relative risk reduction (RRR) in cardiovascular events. This association between achieved LDL-C concentrations and CV risk reduction is continuous, and no final concentration at which benefit ceases has been identified [4, 5].

The benefit of lowering LDL-C also depends on the duration of exposure to high or low plasma concentrations. While data from RCTs show that a 1 mmol/L reduction in LDL-C results in a 22% relative risk reduction (RRR) over about 5 years of intervention, genetic data from Mendelian randomisation studies suggest that maintaining lower LDL-C concentrations for life could yield double the benefit observed in short-term RCTs [6].

Furthermore, in patients who have already experienced a CV event, initiating intensive lipid-lowering therapy early is associated with greater clinical benefits. Recent data based on the prevalence of subclinical atherosclerosis also indicate that LDL-C levels are early determinants of atherosclerosis progression, particularly in younger individuals, suggesting that early intervention (with or without LLT) should be considered promptly [7, 8].

Recent imaging studies have demonstrated that achieving very low plasma LDL-C concentrations not only prevents disease but also promotes plaque healing. The Pacman-AMI, Huygens, and Architect studies [9–11], among others, have shown that reducing LDL-C levels to less than 30 mg/dL results in a significant reduction in the percentage of atherosclerotic plaque mass, cholesterol content, and an increase in the fibromuscular cap, leading to smaller and more stable plaques. Thus, we are transitioning towards a new concept: from atherosclerosis prevention to atherosclerosis treatment [12].

Despite the overwhelming evidence supporting the need to reduce LDL-C concentrations to defined targets, the number of individuals at high and very high CV risk achieving these targets is unacceptably low. Data from the Euroaspire. Da Vinci, and Santorini studies [13–15], among many others, show that only about one-third of patients on LLT achieve the recommended targets. The reasons for this are varied. Although we have therapeutic tools that, when used in combination, can lower LDL-C by more than 80%, they are not adequately prescribed by physicians, and patients tend to discontinue the therapies. Clearly, there is a need to improve treatment by simplifying therapy while maintaining high efficacy.

## What is Inclisiran Adding to Current Lipid-Lowering Therapies?

Statins are the cornerstone of lipid-lowering therapy (LLT) to prevent atherosclerotic cardiovascular disease (ASCVD). There is a substantial body of scientific evidence supporting their use, and there is broad clinical consensus that statins must be the first line of therapy in any LLT regimen.

The use of ezetimibe, a cholesterol absorption inhibitor, is supported by a randomized controlled trial (RCT) (IMPROVE-IT) showing a beneficial effect on cardiovascular event reduction of similar magnitude to that of statins per unit of LDL cholesterol (LDL-C) reduced [16].

Monoclonal antibodies to proprotein convertase subtilisin/kexin type 9 (PCSK9) have demonstrated extremely high lipid-lowering efficacy. Both evolocumab and alirocumab have shown efficacy in reducing cardiovascular events in the FOURIER and ODYSSEY OUTCOMES trials, respectively. However, the proportion of ASCVD risk reduction was somewhat less than expected given the observed LDL-C reduction. This may be due to the early termination of both studies because of clear benefits observed in interim analyses, limiting the observation period to slightly more than two years [17, 18].

Bempedoic acid inhibits cholesterol synthesis at an early step, upstream of HMG-CoA reductase, the target of statins. It is a prodrug activated only in the liver, avoiding activity in peripheral tissues, including muscle. Despite acting in the same pathway as statins, its use is associated with incremental LDL-C reduction when added to statin therapy, with higher efficacy when administered in the absence of statins or with low-dose statins. Recently, the CLEAR Outcomes study demonstrated its efficacy in reducing cardiovascular events in patients intolerant to or tolerant of minimal doses of statins. The magnitude of cardiovascular risk reduction was consistent with the LDL-C lowering achieved [19].

Clinical guidelines recommend very demanding LDL-C targets based on scientific evidence. In patients at high and very high risk, LDL-C should be reduced by at least 50%, achieving levels below 70 mg/dL or 55 mg/dL, respectively [20]. Therefore, monotherapy is generally insufficient, as only high-intensity statins at maximal doses (rosuvastatin 20 mg or atorvastatin 80 mg) reduce LDL-C by 50%. Oral combination therapy (statins plus ezetimibe ± bempedoic acid) can reduce LDL-C by 60–70%, while a combination of oral and subcutaneous therapies (including PCSK9 inhibitors) can reduce LDL-C by more than 80% [12, 20].

Despite this lipid-lowering potential, only a minimum percentage of patients achieve the recommended LDL-C goals.

## What is Inclisiran?

Inclisiran is a novel, first-in-class therapy that targets PCSK9 RNA. This RNA-targeting therapeutic approach represents a new pharmacological strategy compared to traditional methods that inhibit the protein directly. There are two primary mechanisms for RNA interference: antisense oligonucleotide (ASO) and small interfering RNA (siRNA). RNA interference is a naturally occurring physiological mechanism that cells use to regulate gene expression, not an invented technology. Inclisiran leverages this natural mechanism [21, 22].

ASOs are single-stranded nucleotides chain that enter the nucleus and bind to the target RNA, preventing its translation into protein. Inclisiran, on the other hand, is a siRNA composed of a short double-stranded RNA that operates in the cytoplasm. One strand of the siRNA guides the antisense interaction with the RNA-induced silencing complex (RISC), leading to the cleavage of the target mRNA. This process is highly specific because it targets a unique nucleotide sequence, in Inclisiran’s case, the PCSK9 gene. The effect is prolonged because the siRNA remains associated with the RISC complex, continuously blocking mRNA molecules [23].

An important feature of Inclisiran is that the RNA is conjugated to N-acetyl galactosamine (GalNAc), which is recognized by the asialoglycoprotein receptor present exclusively on the surface of hepatocytes. This targeting ensures that Inclisiran is highly specific to the liver.

PCSK9 is primarily synthesized in the liver, though it is also produced in other tissues such as the intestines, pancreas, kidneys, brain, adipose tissue, and vascular cells [24]. PCSK9 is secreted into the circulation and binds to LDL receptors (LDLR), preventing their recycling when internalized. This leads to a reduced expression of LDLR on the hepatocyte surface, diminishing the liver’s ability to internalize LDL particles, which then accumulate in the plasma.

By targeting PCSK9 mRNA, Inclisiran inhibits the hepatic production of the protein, thereby reducing the amount of circulating PCSK9. The absence of PCSK9 in circulation allows LDL receptors to recycle, enhancing LDL clearance by the liver.

A significant difference between Inclisiran and monoclonal antibodies (MoAbs) is that Inclisiran prevents the synthesis of the PCSK9 protein, while MoAbs neutralize the protein after it has been secreted into the circulation. Interestingly, while Inclisiran reduces total circulating PCSK9 levels, MoAb therapies lowers free circulating PCSK9 but increase the total mass of circulating PCSK9 by three to ten-fold by blocking its binding to LDLR, that is its main clearance mechanism [25]. The impact of this fact is not known although it does not appear to have clinical significance.

Furthermore, MoAbs target all circulating PCSK9, while Inclisiran specifically blocks the production of PCSK9 in the liver, which accounts for the major part (70–80%) of the total circulating PCSK9. However, a portion of PCSK9 production outside the liver remains unaffected by Inclisiran, on the other hand, preserving potential extrahepatic functions of PCSK9.

The most important pharmacokinetic (PK) parameters concerning Inclisiran are that its plasma concentrations peak at about 4–6 h after administration, and it takes 9 to 48 h for this drug to disappear from the bloodstream entirely at any given dose [26].

Despite this short half-life, its therapeutic effects last for several months because of the abovementioned mechanism. In contrast, MoAbs remain in circulation to exert their pharmacological action continuously. Table 1 summarizes some of the main pharmacological differences between MoAb and Inclisiran.

**Table 1:** Some pharmacological differences between Inclisiran and anti PCSK9 MoAbs and possible clinical implications

Fact	Alirocumab / Evolocumab	Inclisiran	Putative implications
Type of therapy	Monoclonal Antibody (MoAb)	Small interfering RNA (siRNA)	
Site of action	Plasma	Intracellular in hepatocytes	MoAb targets circulating PCSK9 produced mainly by liver but also by other tissues such as intestine, lung, kidney, pancreas or brain Inclisiran targets hepatic synthesis (70–80% of total) of PCSK9 which might limit its clinical efficacy On the other hand, Inclisiran targets intrahepatic and extracellular functions while MoAb target extracellular functions
LDLc lowering Efficacy	Approximately 50% to > 60% regarding the product and dose	Approximately 45% to 55%	
Plasma Half-life	Days	9–48 Hours	The action of Inclisiran is independent of plasma drug concentrations, while MoAbs must be present in the plasma to be effective. This fact may have safety implications
Duration of pharmacological action	Days to weeks	6 months	Inclisiran’s effect lasts for 6 months without the presence of circulating drug, allowing for longer intervals between doses and sustained and persistent lipid-lowering effects
Impact on circulating PCSK9	Lowers free PCSK9; total plasma concentrations of PCSK9 (bound to MoAb) increase 3- to tenfold	Reduces total plasma concentrations of PCSK9. Some PCSK9 from extrahepatic synthesis remains	Conflicting data exist regarding the relationship between PCSK9 concentrations and cardiovascular risk. The impact of elevated circulating levels of monoclonal antibody-bound PCSK9 is unknown

## What Do We Know About the Clinical Safety and Efficacy of Inclisiran?

The safety and effectiveness of Inclisiran have been thoroughly evaluated in the ORION and VICTORION clinical trial programs, comprising over 25 studies. Phase III randomized controlled trials—ORION-9, ORION-10, and ORION-11—were conducted to assess the safety and efficacy of Inclisiran [27]. These trials involved about 3.660 patients with either atherosclerotic cardiovascular disease (ASCVD) or those at high risk of a cardiovascular event. ORION-9 included patients with heterozygous familial hypercholesterolemia, while ORION-10 and ORION-11 included patients with ASCVD or ASCVD risk equivalents like type 2 diabetes, familial hypercholesterolemia, or a 10-year risk score of 20% or higher [28]. Participants were randomly assigned to receive either 300 mg of Inclisiran sodium or a placebo on Days 1, 90, 270, and 450.

Pooled results from these trials showed that treatment with Inclisiran led to a placebo-corrected decrease in LDL-C of −50.7% by Day 510. A large majority of patients achieved LDL-C levels at or below target goals, with 86.6% reaching levels under 70 mg/dL and 74.6% under 50 mg/dL at any point after baseline. In addition, total cholesterol decreased by 32.4%, non-HDL-C by 46.3%, apoB by 41.7%, Lp(a) by 20.2%, and triglycerides by 9.9%, while HDL-C increased by 6.2% compared to placebo [28]. These studies were extended in ORION-8, an open-label extension of ORION-9, 10, and 11, involving 3,274 patients observed for an average of 36 months [29, 30]. In the long term, Inclisiran reduced LDL by an average of 51% in secondary prevention patients and 42% in primary prevention patients (total group 49.4%). A recent study examining the impact of starting Inclisiran immediately after acute coronary syndrome showed an LDL-C reduction of up to 60% from baseline compared to 7% in the usual care group. More patients receiving "Inclisiran first" achieved LDL-C goals compared to those receiving usual care (< 70 mg/dL: 81.8% vs. 22.2%; < 55 mg/dL: 71.6% vs. 8.9%; *P* < 0.001) [31]. Rates of treatment-emergent adverse events (TEAEs) and serious TEAEs were similar between treatment strategies (62.8% vs. 53.7% and 11.5% vs. 13.4%, respectively). Injection-site TEAEs and TEAEs leading to treatment withdrawal were more common with "Inclisiran first" than with usual care (10.3% vs. 0.0% and 2.6% vs. 0.0%, respectively).

Several meta-analyses focused on the lipid-lowering efficacy of non-statin drugs. A network meta-analysis by Toth et al., which included 48 randomized controlled trials, indicated that Inclisiran was associated with a mean LDL-C reduction of 50 to 52% [32]. Another study by Burnett et al. reviewed 31 studies and found an LDL-C lowering effect of 57% with Inclisiran [33]. There was evidence of variability across the trials, suggesting differences in LDL-C reduction that were generally greater than 5–10%.

In addition to the ORION program, the VICTORION studies are further investigating the long-term efficacy and safety of Inclisiran. VICTORION-1 is focused on high-risk primary prevention patients, while VICTORION-2 and ORION-4 are exploring Inclisiran’s impact in patients with ASCVD [34, 35]. Collectively, these studies include more than 47,000 patients and will provide robust data on the long-term benefits and risks of Inclisiran, complementing evidence from the ORION trials. The VICTORION-PLAQUE study aims to explore Inclisiran’s impact on coronary atherosclerosis burden [36].

Preliminary results from the VICTORION studies suggest that Inclisiran not only maintains its efficacy in lowering LDL-C but also shows promise in reducing the incidence of major cardiovascular events. A subanalysis of pooled data from ORION 9–10–11 focusing on reported adverse events indicated a 25% lower rate of cardiovascular events in the Inclisiran arms (HR: 0.75, 95% CI 0.60–0.94; *p* < 0.013) [37].

Regarding safety, pooled data from ORION 9–10–11 and its open-label extension ORION 8 showed that Inclisiran was generally well tolerated, with similar rates of treatment-emergent events between Inclisiran and placebo groups, except for mild to moderate bronchitis (4.3% vs. 2.7% with placebo; RR 1.55, 95% CI 1.09–2.20) and injection site reactions (5% vs. 0.7% with placebo) [27–29]. These results indicate that Inclisiran effectively reduces LDL-C with minimal variability between patients, demonstrating predictable and consistent efficacy. This predictability is particularly advantageous in chronic therapy, where a stable and expected response to medication is crucial for effective patient management.

The consistent reduction in LDL-C and its impact on cardiovascular outcomes highlight Inclisiran’s potential as a key therapy in managing high-risk cardiovascular patients.

Real-world data from clinical registers provide a closer look at daily clinical practice. Although recently approved for clinical use, several real-world studies, including one at Imperial College London with 80 patients (both FH and non-FH), observed an average LDL-C lowering efficacy of 48.6% two months after initiation, with a favorable safety profile [38]. Similar studies in Italy, Israel and the Netherlands reported mean LDL-C reductions of 49% and 38%, respectively [39, 40]. Another study in Germany with 153 patients showed a mean reduction of 42% in patients on statins, whereas efficacy was 23% in patients previously on PCSK9 inhibitors [41]. Across these studies, the safety profile was generally good, with injection site reactions being the most frequent adverse event [38–41]. Table 2 summarize the aims and main information provided by the phase III Inclisiran RCT.

**Table 2:** Phase III randomized controlled trials with Inclisiran and their main clinical implications

Trial	Population	Endpoint/Results
ORION-10 and ORION-11	ASCVD or ASCVD risk equivalents	LDL-C reduction: 50% (ORION-10), 52% (ORION-11)
ORION-9	HeFH or ASCVD with elevated LDL-C despite LLTs	LDL-C reduction: 49%
ORION-4	ASCVD or ASCVD risk equivalents with high LDL-C	LDL-C reduction: 50%
ORION-18	Asian patients with ASCVD or high risk of ASCVD	≥ 50% LDL-C reduction in 71.7% of participants
ORION-2	Homozygous Familial Hypercholesterolemia HoFH	Early phase II study contributing to understanding Inclisiran’s efficacy
ORION-5	Homozygous Familial Hypercholesterolemia HoFH	Phase III study contributing to safety and efficacy profile
ORION-6 and ORION-7	Liver and kidney disease subgroups	Phase I studies contributing to cumulative data on Inclisiran’s effectiveness
ORION-8	Patients from ORION-9, ORION-10, ORION-11, and ORION-3	Sustained LDL-C reduction over 3 years, favorable safety profile
ORION-13	Adolescents with HoFH and elevated LDL-C	Ongoing; early data suggests significant LDL-C reduction and manageable safety profile
ORION-14	Chinese participants with elevated LDL-C despite LLTs	Effective LDL-C reduction with good tolerability
ORION-15	Japanese patients with high cardiovascular risk and LDL-C	Significant LDL-C reduction consistent with global findings
ORION-16	Adolescents with HeFH and elevated LDL-C	Ongoing; expected to confirm efficacy and safety in younger populations
VICTORION-1 PREVENT	High-risk patients without previous major cardiovascular events	25% reduction in MACE compared to placebo
VICTORION-2 PREVENT	Patients with established cardiovascular disease	24% reduction in MACE compared to placebo
VICTORION-PLAQUE	Patients with non-obstructive coronary artery disease	Significant reduction in total coronary plaque volume
VICTORION-INCEPTION	Post-acute coronary syndrome patients	Significant LDL-C reduction within a year, aiming for levels below 70 mg/dL
VICTORION-INITIATE	ASCVD patients	Ongoing; comparing ‘Inclisiran first’ strategy with usual care
VICTORION-MONO	Patients with primary hypercholesterolemia	Significant LDL-C reduction compared to placebo and ezetimibe; evaluated as monotherapy
VICTORION-SPIRIT	Primary care patients in the UK	Superior LDL-C reduction compared to standard care; potential to change lipid management approach in healthcare systems

### Up to Twenty-Two Injections Avoided Each Year

Additional advantages of Inclisiran include its persistent LDL-C lowering effect due to its long-lasting action and increased adherence. Inclisiran’s twice-yearly dosing schedule simplifies therapy compared to anti-PCSK9 monoclonal antibodies, which require injections every two to four weeks and often must be administered in hospital settings. This difference not only affects patients but also impacts healthcare systems, requiring coordinated care across various healthcare levels.

## When and How Including Inclisiran in the LLT Strategy?

The 2019 clinical guidelines from the European Society of Cardiology and European Atherosclerosis Society recommend a step-by-step approach to implementing lipid-lowering therapy (LLT) [20]. This approach begins with using statins alone initially, and then adding non-statin drugs sequentially if treatment goals are not met. The suggested sequence includes statins, ezetimibe, bempedoic acid, and therapies targeting PCSK9.

However, this sequential strategy is time-consuming, requiring more than 2 to 3 months to assess the effectiveness of each step. Often, adjustments are discontinued after a few months or patient follow-up is lost, resulting in a low rate of patients achieving the recommended LDL cholesterol targets [13–15].

Conversely, it is recognized that achieving lower LDL cholesterol levels is crucial, especially for patients at high or very high cardiovascular risk, and earlier intervention is beneficial. Therefore, there is advocacy for utilizing combination therapies from the outset, incorporating all available tools. One proposed alternative strategy involves planning therapy by prescribing the necessary drug or combination to approach the target LDL cholesterol level from the beginning. Some experts suggest that in very high-risk patients, maximum LDL cholesterol reduction should be aimed for from the start [42–44].

Inclisiran is recommended for inclusion in high-intensity therapies, particularly for very high-risk patients. Table 3 illustrates the potential LDL cholesterol reduction capacity achievable with combination therapies that include Inclisiran. For instance, combining a high-intensity statin with ezetimibe and Inclisiran can reduce LDL cholesterol by more than 80%, enabling achievement of target levels even for patients with a baseline LDL cholesterol up to 300 mg/dl [12, 44].

**Table 3:** Theoretical LDLc reduction (%) to be obtained with lipid lowering drug combinations including Inclisiran

	Theoretical LDLc reduction (%)	Maximal baseline value that could achieve the objective C-LDL < 70 mg/dl	Maximal baseline value that could achieve the objective C-LDL < 55 mg/dl
Inclisiran + Ezetimibe	64	194	153
Inclisiran + Bempedoic	65	200	157
Inclisiran + Ezetimibe + Bempedòic	72	250	196
Inclisiran + Statin MI	73	259	203
Inclisiran + Statin HI	78	318	220
Inclisiran + Statin MI + Ezetimibe	78	318	220
Inclisiran + Statin HI + Ezetimibe	82	388	305
Inclisiran + Statin MI + Ezetimibe + Bempedoic	84	437	343
Inclisiran + Statin HI + Ezetimiba + Bempedoic	85	466	367

*MI* Moderate Intensity, *HI* High Intensity

Generally, as with other PCSK9 inhibitors, the decision to include Inclisiran in the treatment regimen should be made no later than 2 months after initiating oral high-intensity LLT. However, beginning with higher-intensity strategies from the outset should also be considered.

The Inclisiran first study has demonstrated the benefits of initiating combination therapy with a statin and Inclisiran immediately after myocardial infarction (MI). This approach could be a viable alternative, ensuring persistent high-intensity LLT during a critical period for patients at very high risk [31].

## Conclusion

Inclisiran is a novel drug offering high-intensity lipid-lowering efficacy, a favorable safety profile, and a convenient administration regimen, which should enhance adherence and persistently maintain low LDL cholesterol levels over time.

## Key References

- Ray KK, Haq I, Bilitou A, Manu MC, Burden A, Aguiar C et al. SANTORINI Study Investigators. Treatment gaps in the implementation of LDL cholesterol control among high- and very high-risk patients in Europe between 2020 and 2021: the multinational observational SANTORINI study. Lancet Reg Health Eur. 2023 Apr 5;29:100624. 10.1016/j.lanepe.2023.100624.
  - The use of lípid lowering therapies is unappropriated and the achievement of targets is too low.
- Kulkarni JA, Witzigmann D, Thomson SB, Chen S, Leavitt BR, Cullis PR et al. The current landscape of nucleic acid therapeutics. Nat Nanotechnol. 2021 Jun;16(6):630–643. 10.1038/s41565-021-00898-0.
  - Review of new siRNA therapies. Inclisiran mimicks cell physiology.
- Koenig, W. et al. Efficacy and Safety of Inclisiran in Patients with Polyvascular Disease: Pooled, Post Hoc Analysis of the ORION-9, ORION-10, and ORION-11 Phase 3 Randomized Controlled Trials. Cardiovasc Drugs Ther (2022) 10.1007/s10557-022-07413-0.
  - Pooled data from Orion 9, 10, 11 showing the efficacy and safety of Inclisiran in different clinical conditions
- Wright, R. S. et al. Inclisiran administration potently and durably lowers LDL-C over an extended-term follow-up: the ORION-8 trial. Cardiovasc Res (2024) 10.1093/cvr/cvae109.
  - Extended study of Inclisiran evaluating its long term effects
- Koren MJ, Rodriguez F, East C, Toth PP, Watwe V, Abbas CA et al. An "Inclisiran First" Strategy vs Usual Care in Patients With Atherosclerotic Cardiovascular Disease. J Am Coll Cardiol. 2024 May 21;83(20):1939–1952. 10.1016/j.jacc.2024.03.382.
  - Starting Inclisiran from the outset improves the lipid lowering yield
- Burnett H, Fahrbach K, Cichewicz A, Jindal R, Tarpey J, Durand A et al. Comparative efficacy of non-statin lipid-lowering therapies in patients with hypercholesterolemia at increased cardiovascular risk: a network meta-analysis. Curr Med Res Opin. 2022 May;38(5):777–784. 10.1080/03007995.2022.2049164.
  - Meta-analysis stablishing the efficacy of non-statin lipid lowering therapies comparing PCSK9 targeted therapies.
- Ray KK, Reeskamp LF, Laufs U, Banach M, Mach F, Tokgözoğlu LS et al. Combination lipid-lowering therapy as first-line strategy in very high-risk patients. Eur Heart J. 2021 Oct 12:ehab718. 10.1093/eurheartj/ehab718.
  - In patients at very high CV risk strategies the maximum LLT capacity from the outset are recommended

## Data Availability

No datasets were generated or analyzed during the current study.
